# Hantavirus co-circulation in common shrews (*Sorex araneus*) in Sweden

**DOI:** 10.1093/ve/veaf038

**Published:** 2025-05-28

**Authors:** Anishia Wasberg, Frauke Ecke, Johanna F Lindahl, John H-O Pettersson, Åke Lundkvist, Jiaxin Ling

**Affiliations:** Zoonosis Science Center, Department of Medical Biochemistry and Microbiology, Uppsala University, Husargatan 3, 752 37 Uppsala, Sweden; Department of Wildlife, Fish, and Environmental Studies, Swedish University of Agricultural Sciences, Skogsmarksgränd, 901 83 Umeå, Sweden; Organismal and Evolutionary Biology Research Programme, University of Helsinki, PO Box 65, 00014 Helsinki, Finland; Zoonosis Science Center, Department of Medical Biochemistry and Microbiology, Uppsala University, Husargatan 3, 752 37 Uppsala, Sweden; Department of Animal Health and Antibiotic Strategies, Swedish Veterinary Agency, Ulls väg 2B, 751 89 Uppsala, Sweden; Zoonosis Science Center, Clinical Microbiology, Department of Medical Sciences, Uppsala University, Husargatan 3, 752 37 Uppsala, Sweden; Department of Microbiology, Swedish Veterinary Agency, Ulls väg 2B, 751 89 Uppsala, Sweden; Department of Microbiology and Immunology, Peter Doherty Institute for Infection and Immunity, University of Melbourne, 792 Elizabeth Street, Melbourne VIC 3000, Australia; Zoonosis Science Center, Department of Medical Biochemistry and Microbiology, Uppsala University, Husargatan 3, 752 37 Uppsala, Sweden; Zoonosis Science Center, Department of Medical Biochemistry and Microbiology, Uppsala University, Husargatan 3, 752 37 Uppsala, Sweden

**Keywords:** hantavirus, insectivores, Seewis virus, Altai virus, RNA sequencing, phylogeny

## Abstract

Shrews are primary hosts for mammalian hantaviruses and are thus considered to be important reservoirs for viruses, similar to rodents and bats. To explore the diversity of hantaviruses in Swedish common shrews (*Sorex araneus)*, we investigated lung tissue from shrews collected between 2015 and 2017. The collection took place at three separate locations in south-central Sweden. Screening for hantaviruses was performed using two different approaches. (i) A total of 113 common shrews were investigated for hantaviruses by a pan-hantavirus L-gene reverse transcriptase PCR, and Sanger sequencing was performed on the 13 positive samples. (ii) In addition, 88 RNA samples were pooled into eight libraries subjected to RNA sequencing. The RNA sequencing data analysis, which focused specifically on identifying hantaviruses, revealed two divergent hantaviruses: the complete genome of an Altai virus (ALTV) and the partial genome of the Seewis virus. Evolutionary analysis revealed that Swedish ALTVs are closely related to Russian ALTVs but distinct from Finnish strains. On the contrary, the Swedish Seewis virus shares closer ancestry with Finnish Seewis virus strains. Given that these viruses were identified in several pools, Seewis virus and ALTV are likely circulating in Swedish common shrews. Supported by earlier studies, common shrews are probably a natural host for at least these two distinct hantaviruses.

## Introduction

Hantaviruses (order *Elliovirales* and family *Hantaviridae*; Bradfute *et al*. 2024) are enveloped, trisegmented, single-stranded, negative-sense RNA viruses. Hantaviruses in the *Mammantavirinae* subfamily are mainly carried by rodents, but shrews, moles, and bats are important reservoir animals. The subfamily can be further classified into four different genera: *Orthohantavirus*, *Loanvirus*, *Mobatvirus*, and *Thottimvirus*, where the three latter hantavirus genera were established after DivErsity pArtitioning by hieRarchical Clustering (DEmARC) was selected as the preferential method for classification of novel divergent hantaviruses discovered in Eulipotyphla and bats (Laenen *et al*. 2019). Some rodent-borne hantaviruses infect humans and cause disease, e.g. haemorrhagic fever with renal syndrome (HFRS), nephropathia epidemica (NE, a milder form of HFRS), and hantavirus pulmonary syndrome (Guo *et al*. 2013). Knowledge of the zoonotic potential of hantaviruses from unconventional hosts such as shrews, moles, and bats is lacking, although there is serological evidence that shrew-borne hantaviruses might infect humans (Heinemann *et al*. 2016).

In Sweden, hantaviruses have mainly been explored in rodents, with a focus on Puumala orthohantavirus (PUUV), carried in particular by the bank vole (*Clethrionomys glareolus*), with prevalence generally being higher in bank voles trapped in spring compared to autumn (Niklasson *et al*. 1995, Nemirov *et al*. 2010, Khalil *et al*. 2016, 2019, Borg *et al*. 2017). PUUV is the causative agent of NE, the only known hantavirus-caused clinical infection in Sweden (Olsson *et al*. 2009, Nemirov *et al*. 2010, Vaheri *et al*. 2013). Although most clinical cases of NE are clustered in northern Sweden, accumulating evidence indicates that there may be a greater genetic diversity and a broader geographical distribution of hantaviruses in Sweden than previously recognized. For example, several serological studies revealed that PUUV-infected rodents are also present south of the hitherto acknowledged endemic areas of PUUV in northern Sweden (Lõhmus *et al*. 2016, Borg *et al*. 2017). Notably, the first locally acquired NE case in southernmost Sweden was reported in 2018, in an area with no prior documented hantavirus infections (Elin and Frisk 2019, Ling *et al*. 2024). An additional NE case emerged in the same region in 2020. These results suggest that there may be new variants of hantaviruses circulating in known and previously unidentified host reservoirs and geographical areas in Sweden.

The common shrew (*Sorex araneus*) has been suggested to be the natural host for Seewis hantavirus (SWSV), the prototype shrew-borne hantavirus in Europe. SWSV was first discovered in the Canton of the Grisons, Switzerland, in 2007 (Song *et al*. 2007) and later in Finland, Poland, Russia, Germany, and Sweden (Kang *et al*. 2009, 2019, Schlegel *et al*. 2012, Lwande *et al*. 2020). Furthermore, common shrews have also been found to carry divergent Altai viruses (ALTVs) (Ling *et al*. 2014, Kang *et al*. 2019), suggesting that this common shrew species can carry more than one hantavirus species. Currently, there are no Seewis or Altai hantavirus isolates and no full-genome sequence of SWSV is available. This study aimed to investigate the diversity of hantaviruses circulating in Swedish common shrews by applying an integrative approach that combined conventional methods (RT-PCR and Sanger sequencing) with RNA sequencing. This strategy allowed for the detection of co-circulating hantaviruses and enabled full-genome characterization of the identified viruses. The genomic sequences of shrew-borne hantaviruses will provide important insights into the evolution, ecology, and possible differences in pathogenicity between rodent-borne hantaviruses.

## Method and materials

### Sample collection

All trapping and sampling were approved by the Animal Ethics Committee in Umeå (Reference: A 13–14, A 39–14), and the Swedish Environmental Protection Agency (Reference: NV-01124-15). All applicable institutional and national guidelines for the use of animals were followed.

**Figure 1:**
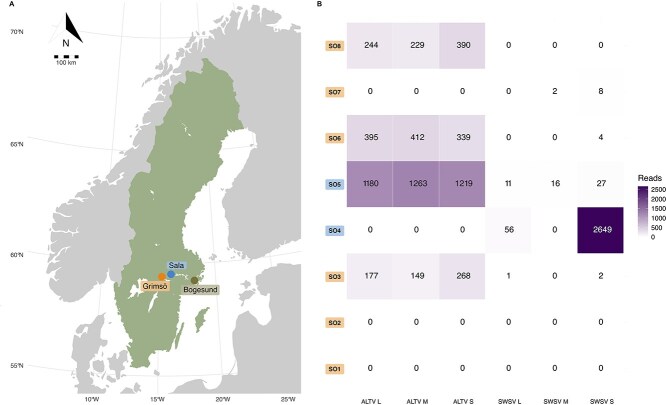
(A) Map showing the location of the three sampling sites: Grimsö, Sala and Bogesund. The five samples from Bogesund were negative for hantaviruses. (B) Heat map depicting the number of reads mapped to each viral segment for both ALTV and SWS throughout the generated pools SO1, SO2, SO3, SO4, SO5, SO6, SO7, and SO8. Pools SO1, SO2, SO3, SO6, SO7, and SO8 are from Grimsö; SO4 and SO5 are from Sala.

In this study, rodents and shrews were snap-trapped at three different locations, viz. Grimsö (59°43′ N, 15°28′ E), Bogesund (59°24′ N, 18°14′ E), and a major (130 km^2^) wildfire area near Sala, affected by a wildfire in 2014 (59°54′ N, 16°09′ E), in Sweden between May 2015 and November 2017 (Fig. 1A). The captured animals were kept at −20°C within 2 h after checking the traps and before transferring the animals to the laboratory. Specimens were identified at the species level in the laboratory and dissected immediately, where lung tissue was collected and subsequently frozen at −80°C until analyses.

After initial morphological identification, 113 specimens were identified as common shrews (*S. araneus*) and 27 as pygmy shrews (*Sorex minutus*). To confirm the species, total DNA was extracted from the lung tissue using the DNeasy Blood & Tissue Kit (Qiagen, Hilden, Germany), followed by sequencing of the cytochrome-b gene, as described earlier (Kang *et al*. 2009). Based on the sequencing results, 110 samples were confirmed as common shrews (Supplementary Table S1).

### PCR-screening for hantaviruses

A nested pan-hantavirus L-gene RT-PCR developed by Klempa and co-workers (Klempa *et al*. 2006) was used to search for hantaviruses in all 140 shrew samples. Briefly, total RNA from lung tissue was extracted using the Qiagen RNeasy Mini Kit (Qiagen, Hilden, Germany). Subsequently, cDNA was synthesized using the SuperScript III Reverse Transcriptase (Invitrogen by Thermo Fisher Scientific, CA, USA), whereafter the nested PCR was performed using Phusion Flash High-Fidelity PCR Master Mix (Thermo Fisher Scientific, MA, USA). All pan-hantavirus L-gene PCR products were Sanger sequenced at Macrogen Europe (https://www.macrogen-europe.com).

### RNA-sequencing

To minimize variation in sample representation across pools and improve comparability in sequencing results, in total, 88 out of 110 RNA samples confirmed from *S. araneus* were pooled into eight pools, each pool having 11 RNA samples from the same location. Six pools (SO1, SO2, SO3, SO6, SO7, and SO8) were from Grimsö and two pools (SO4 and SO5) were from Sala. The remaining 22 samples were excluded to maintain consistent pool sizes across sampling sites (Supplementary Table S1). Subsequently, eight RNA-sequencing (RNA-Seq) libraries were constructed using the KAPA RNA HyperPrep with RiboErase library prep kit (Roche, Basel, Switzerland). The constructed libraries were quality checked on a TapeStation from Agilent Technologies (Santa Clara, CA, USA) before paired-end 150 bp sequencing was performed using the Illumina Xten sequencing platform at BGI Genomics (https://www.bgi.com/global/). The number of raw reads reached for each library was around 50 M.

The initial data analysis pipeline was used as described in a previous study (Ling *et al*. 2020). In brief, contigs were obtained through *de novo* assembly of quality-checked reads using Trinity v.2.5.4 (Grabherr *et al*. 2011). Later, *de novo* assembled contigs larger than 200 bp were screened using BLASTN v.2.6.0+ and Diamond v.0.9.15.116 (Buchfink *et al*. 2021) against the complete NCBI non-redundant nucleotide and protein databases with 1 × 10^−5^ as a cut-off *e*-value. Sequence contigs that were indicated to match hantavirus reference sequences were pulled out from each sequence library by using seqtk v.1.2 (https://github.com/lh3/seqtk). All hantaviral-like contigs were then verified using BLASTN. Then, the most complete S, M, and L contigs for each virus (variant) were mapped using the Burrows–Wheeler Aligner v.0.7.13 to estimate sequence depth and verify the consensus sequence. All sequences generated in this study have been deposited in NCBI GenBank with accession numbers ON720814–ON720835 and PP578938–PP578951.

### Phylogenetic analyses

To reconstruct the evolutionary history of SWSV and ALTV, three hantavirus multiple sequence alignments were prepared for each virus: one S-segment alignment, one M-segment alignment, and one L-segment alignment, each including all previously published SWSV or ALTV sequences along with contigs from the RNA-Seq data. This was done for both nucleotide and amino acid sequences. Additionally, multiple sequence alignments were prepared for partial-L sequences retrieved from the conventional RT-PCR together with 130 partial-L sequences retrieved from the NCBI. All these datasets were aligned using MAFFT v.7 with default settings (Katoh and Standley 2013), followed by manual refinement using AliView (Larsson 2014) and removal of all positions with 10% gaps using trimAl v.1.4.1 (Capella-Gutiérrez *et al*. 2009). Potential recombination events were investigated using RDP3 (Martin *et al*. 2010). The analysis included the application of seven different recombination detection methods implemented in the software: RDP, GENECONV, BOOTSCAN, MaxChi, Chimaera, SiScan, and 3Seq. Default settings were used for analysing linear sequences.

To analyse the phylogenetic relationship of the hantavirus sequences produced in the present study in relation to other hantaviruses worldwide, phylogenetic trees were constructed using MrBayes v.3.2.7a (Ronquist and Huelsenbeck 2003). The best-fit model was determined by jModelTest v.3.7 (Posada 2008), whereafter the GTR + F + I + G4 model of nucleotide evolution was used in all phylogenetic analyses. The Bayesian analysis consisted of at least 5 million Bayesian Monte Carlo Markov chain generations, sampling every 1000 generations. The run continued until convergence (average deviation, <0.01), followed by a 25% burn-in. For amino acid tree construction, the fixed standard WAG +I + G4 (Whelan and Goldman 2001) model of protein evolution was used with the remaining settings the same, as previously mentioned. All the phylogenetic results were visualized in FigTree (version 1.40). All the computations were run using the UPPMAX computational cluster (https://www.uppmax.uu.se/). Final data rendering and visualization were performed in RStudio (version 1.4.1106) and Geneious Prime 2024.0.7 (https://www.geneious.com).

## Results

### Prevalence of hantaviruses in Swedish common shrews

Using a conventional RT-PCR screening for hantaviruses based on the RNA-dependent RNA polymerase region, or the L-segment, we found 13 hantavirus-positive samples out of 110 samples confirmed to be from *S. araneus*. Partial L-segment sequences (254 bp) were generated from 11 samples through Sanger sequencing. All the positive samples originated from autumn trappings, and the distribution of positive samples was as follows: 0/5 in Bogesund, 2/30 in Sala, and 11/75 in Grimsö. The NCBI standard nucleotide BLAST of the 11 L-segment sequences revealed that all were SWSV sequences.

## RNA-Seq for hantavirus

**Figure 2:**
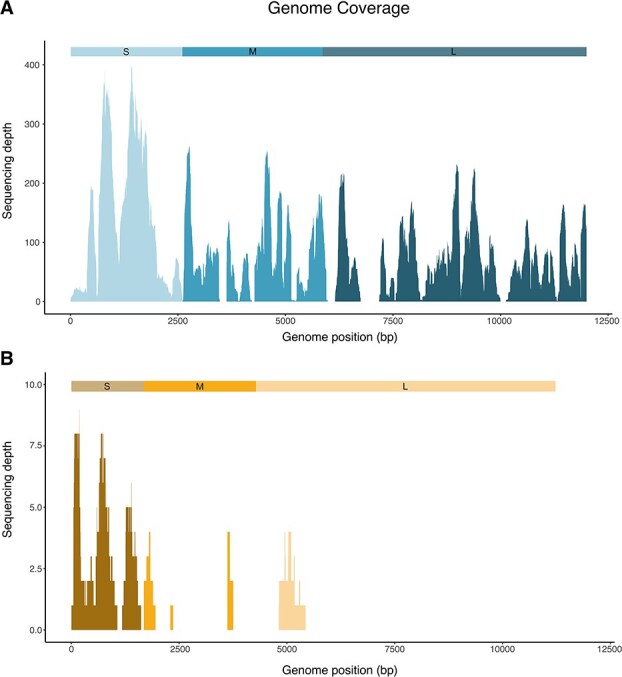
(A) Genome coverage and sequencing depth of Swedish ALTV and (B) SWSV S-, M-, and L-sequences retrieved from pool SO5 by *de novo* sequencing.

The RNA-Seq generated ~50 M reads per library for all eight pooled libraries. Following *de novo* assembly and screening, we identified two hantaviruses in our eight libraries: ALTV and SWSV. Specifically, we obtained complete genome sequences of an ALTV, which comprised a 2016 nt S-segment, 3575 nt M-segment, and 6517 nt L-segment. In addition, we obtained ~55% of the SWSV genome, comprising 1621/1641 nt for the S-segment, 972/3533 nt for the M-segment, and 3856/6571 nt for the L-segment. The genome coverage for ALTV (L-, M-, and S-segments) and SWSV (M- and S-segments) references are shown in Supplementary Fig. S1. The number of hantavirus reads found in each library for ALTV and SWSV is shown in Fig. 1B. ALTV was found in four libraries and SWSV was found in two libraries, where both SWSV and ALTV were present in library SO5 (Fig. 1B). Furthermore, in SO5, a similar number of reads were mapped to each SWSV segment (11/16/27, No. of reads for the L-, M-, and S-segments); the same pattern could be observed for ALTV (1180/1263/1219, No. of reads mapped to the L-, M-, and S-segments). Figure 2 shows the raw read coverage of full-length ALTV sequences and SWSV sequences generated through *de novo* assembly, highlighting the prominent difference in sequence coverage between ALTV and SWSV.

### Phylogeny of Swedish SWSV and ALTV

**Figure 3:**
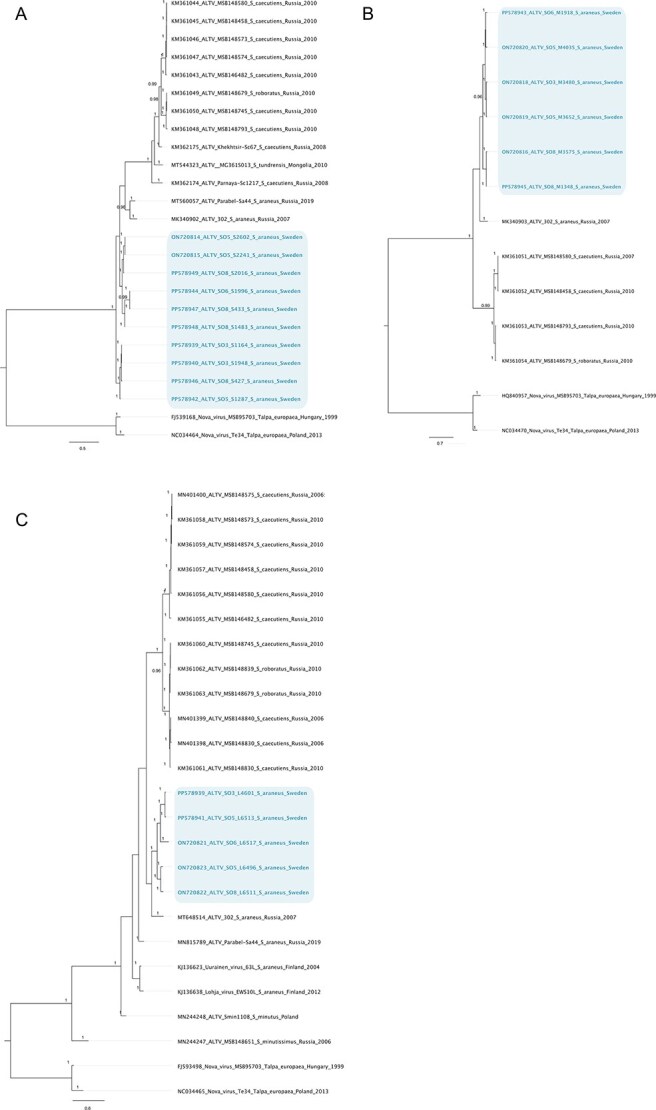
Bayesian phylogenetic trees of Swedish ALTV based on (A) S-segment, (B) M-segment, and (C) L-segment nucleotide alignments. The final alignment lengths were 930 nt (S-segment), 2161 nt (M-segment), and 2375 nt (L-segment). Each alignment also includes shorter partial sequences to maximize the representation of available ALTV diversity. Sequences generated in this study are highlighted in colour. Phylogenetic reconstruction was performed using MrBayes under the GTR + G substitution model. Posterior probability values are shown at nodes, and scale bars indicate nucleotide substitutions per site.

To understand the evolutionary relationships of Swedish ALTV and SWSV with other hantaviral relatives, phylogenetic trees based on hantavirus genome segments were reconstructed. After trimming, the final alignment lengths for the ALTV segments were as follows: The **L-segment** alignment covered 2375 nt but also included a number of shorter partial sequences: two Finnish sequences (282 nt), one Polish sequence (769 nt), and nine Russian sequences (≥282 nt). For the **M-segment**, the alignment spanned 2161 nt and similarly included two shorter Swedish sequences (≥1348 nt) and four shorter Russian sequences (≥460 nt). The **S-segment** alignment totalled 930 nt, incorporating four shorter Swedish sequences (≥338 nt) and five shorter Russian sequences (≥553 nt). These shorter sequences were included to increase the representation of geographically and genetically informative strains (Supplementary Table S2). For ALTV, phylogenetic analysis indicates the presence of at least three phylogroups of ALTV, and that all Swedish ALTV segments (L, M, and S) have a close relationship with ALTV from Russia (variant ALT 302). For the L segment, Russian and Finnish sequences are available, which disclosed a more distant relationship to Finnish Uurainen and Lohja virus strains compared to Russian ALTV strains (Fig. 3). The average pairwise identity for all globally available ALTV L-segment sequences was 76.7% (IQR 74.8%–84.1%) at the nucleotide level and 82.1% (IQR 82.8%–97.5%) at the amino acid level. Within Swedish ALTV sequences, the average pairwise identity ranged from 84.2% to 85.2% at the nucleotide level and 98.5% to 98.9% at the amino acid level (Supplementary Fig. S2).

**Figure 4:**
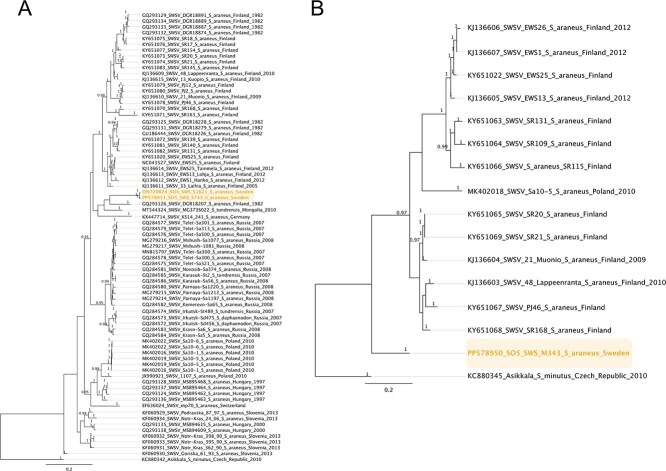
Bayesian phylogenetic trees of Swedish Seewis virus (SWSV) based on (A) S-segment and (B) M-segment nucleotide alignments. The final alignment lengths were 786 bp for the S-segment and 1187 bp for the M-segment. Both alignments included shorter partial sequences to capture the genetic diversity and geographical distribution of SWSV. Sequences generated in this study are highlighted in colour. Phylogenetic reconstruction was performed using MrBayes under the GTR + G substitution model. Posterior probability values are shown at nodes, and scale bars indicate nucleotide substitutions per site. The Swedish SWSV sequences cluster closely with Finnish SWSV strains, forming a distinct clade.

Comparing the nucleotide pairwise identity between Swedish, Russian, and Finnish L-segments, the pairwise identity between Swedish and Russian sequences ranged from 75.2% to 84.7% at the nucleotide level, and 90.6%–98.5% at the amino acid level and the pairwise identity between Swedish and Finnish ALTV ranged from 78.4% to 84.1% at the nucleotide level and 95.7%–98.7% at the amino acid level. The two Finnish sequences included in this analysis are only 282 bp long (94 aa) and align to conserved regions of the Swedish L segment, which explains the high amino acid pairwise identity.

On the other hand, the phylogeny of Swedish SWSV suggested a different evolutionary pathway for SWSV compared to ALTV, supported by the topology of SWSV M and S sequences (Fig. 4). For the Seewis virus (SWSV), the final alignment lengths after trimming for the **M-segment** were 1187 nt and included a number of shorter partial sequences: one Swedish sequence (343 nt), two Finnish sequences (≥847 nt), and one Polish sequence (794 nt). The **S-segment** alignment was 786 nt and also included shorter sequences: one Russian sequence (510 nt), three Polish sequences (≥619 nt), five Hungarian sequences (≥394 nt), seven Finnish sequences (≥394 nt), and one Swedish sequence with 670 nt (Supplementary Table S2). Here, the M- and S-segments of Swedish SWSV shared a most recent common ancestry with SWSV from Finland. The phylogeny of the SWSV L-segment was not included due to no, or poor, alignment between Swedish L-segment sequences and other available L-segments at both nucleotide and amino acid levels. However, the phylogeny based on 11 partial sequences of the SWSV L-segment obtained from conventional PCR screening revealed a supported shared ancestry between Swedish and Finnish SWSV viruses (Supplementary Fig. S4). Additionally, the average pairwise identity of the obtained 11 partial sequences, together with 130 partial L-segments, was at 81.9% (IQR 79.6%–84.2%) at the nucleotide level and ranged from 80.2% to 97.3% at the amino acid level. Within the Swedish sequences, the identity of SWSV ranged from 91.7% to 94.9% at the nucleotide level and 96.4%–100% at the amino acid level. Compared with SWSV from other countries, Swedish SWSV was closer to the Finnish SWSV (IQR 83.0%–88.7% at nucleotide level and 81.1%–97.6% at amino acid level), based on 289 bp of sequences. Amino acid trees of ALTV and SWSV S-, M-, and L-segments are depicted in Supplementary Fig. S3.

Additionally, analysis of potential reassortment and recombination between ALTV and SWSV showed no such events have occurred. The absence of detectable reassortment among the Swedish ALTV and SWSV strains, as indicated by the congruent phylogenetic trees and consistent level of reads from three segments of two viruses, suggests that these viruses may primarily evolve through accumulation of mutations rather than through segment exchange. However, given that reassortment has been described for other shrew-borne hantaviruses (Lee *et al*. 2017, Ling *et al*. 2018) its occurrence cannot be entirely excluded without more extensive sampling and deeper genome coverage.

## Discussion

This study provides the first report of ALTV in Sweden. The first ALTV sequence was found in the Altai Republic, near Lake Teletskoye, Russia, in 2007, following a hantavirus screening of *S. araneus* (Yashina *et al*. 2010). Later, two Altai sequences were found in common shrews in Finland (Ling *et al*. 2014). Although additional sequence data on ALTVs have been obtained recently (Kang *et al*. 2019), the diversity of this virus still needs to be determined. In 2020, a proposed Altai full-length prototype virus was uploaded to NCBI GenBank (Yashina *et al*. 2021). Our study, by using RNA sequencing, successfully recovered the full-genome of the ALTV, which can help taxonomically assign ALTVs into a new species in the genus *Mobatvirus* within the subfamily *Mammantavirinae*.

By combining conventional RT-PCR and Sanger sequencing with high-throughput RNA-Seq, this study successfully characterized the genetic diversity and phylogenetic relationships of co-circulating hantaviruses, including the first detection of ALTV in Sweden. By using conventional pan L-hanta RT-PCR screening, the targets were more skewed towards SWSV or closer relatives, since no ALTV was detected by this method in our study; despite this, the currently available ALTV sequences were discovered through the same method (Kang *et al*. 2019). By using RNA-Seq of RNA derived from lung tissue from common shrews in Sweden, we detected both SWSV and ALTV; ALTV was found in four pools, while SWSV was found in two pools and prominent. Common across the pools was the read abundance for ALTV and SWSV, where the raw read coverage for ALTV was higher and more complete than that of SWSV. This might be due to the fact that our study focused on lung tissue, whereas the results would differ if SWSV and ALTV had different lung tissue tropisms. This might also explain why previous isolation attempts of SWSV failed when using lung tissue (Ling, unpublished results); on the contrary, retrieving ALTV isolates from lung tissue of the common shrews seems promising. SWSV RNA has previously been detected in lung, kidney, and liver tissues (Hönig *et al*. 2022, Karić *et al*. 2023); however, comprehensive studies on the full spectrum of tissue tropism in shrews are still limited. All these accumulated examples suggest that *S. araneus* is carrying ALTV, in addition to SWSV. In one library, SO4, the SWSV sequencing data displayed a prominent disparity in the distribution of reads mapping to the L-, M-, and S-segments. Specifically, we observed 56 reads mapped to the L-segment, no reads mapped to the M-segment, and over 2000 reads mapped to the S-segment. This skewed distribution could result from differential expression of the viral segments within the host cells during infection.

Understanding the enigmatic genealogy of SWSV and ALTV could also help in understanding the evolution of hantavirus. How these two viruses came to co-circulate in the same host species is still unknown. However, based on the phylogeny of SWSV and ALTV from this study, we hypothesize that these two distinct hantaviruses have different evolutionary histories, where ALTV in Sweden has an origin in the Russian/Siberian phylogroup, while SWSV in Sweden was introduced from the eastern Carpathian phylogroup (Raspopova *et al*. 2020). By including the calibration of biogeographical events in Fennoscandia, the evolutionary analysis can be determined, similar to what has previously been described for PUUV (Razzauti *et al*. 2009) and by Plyusnin and Sironen (2014) or Pettersson and Fiz-Palacios for viruses from the *Flavivirus* genus (Pettersson and Fiz-Palacios 2014), and such inference requires demographical history and the evolutionary history of common shrews during the last glacial maximum, as well as more sequences of SWSV and ALTV being available. Without more data on the viruses or hosts, in this study, we cannot test further our hypothesis on the evolutionary trajectories of these two viruses, wherein we propose that SWSV, together with *S. araneus,* was recolonised to Fennoscanidia during the late Pleistocene from the Carpathian refugium, while ALTV together with *S. araneus* already existed before that time period.

The co-circulation of the two hantaviruses allowed us to test whether inter-genera reassortment and within-species recombination events have taken place. We did not find evidence for any reassortment or recombination events between and within SWSV and ALTV. However, SWSV has previously been reported to have undergone reassortment events (Ling *et al*. 2018). In the SO5 library, both SWSV and ALTV were found. Although they indicated the presence of both viruses, the number of reads mapping to ALTV L-, M-, and S-segments was considerably higher than those for SWSV. These results indicated that the likelihood of a reassortment event was very low. Taken together, our sequencing results indicated that the co-circulation of SWSV and ALTV occurs in the form of two intact viruses.

The confirmation that two distinct hantaviruses are circulating in the same host species supports the role of insectivorous mammals as natural hosts for mammalian pathogenic hantaviruses and other zoonotic RNA viruses (Chen *et al*. 2023), such as Hepatitis B viruses found in shrews (Rasche *et al*. 2019).

Although we trapped common shrews in both the spring and autumn, all the positive samples were derived from common shrews trapped in the autumn. This raises the question of the different seasonal dynamics of the hantaviruses identified here. It is known that other hantaviruses display seasonal patterns. For example, PUUV, which has bank voles as a reservoir, usually shows a higher prevalence in spring (Voutilainen *et al*. 2016, Khalil *et al*. 2016, 2019). In our study, however, only seven samples were from shrews trapped in spring and this low sample size could explain the absence of the virus in our study.

Shrews are Soricomorpha species and can act as reservoirs for different zoonotic infections. Here, we provide evidence that Swedish common shrews are likely the natural host for ALTV in addition to SWSV hantavirus, most likely with distinct evolutionary and geographical histories. Therefore, it is of utmost importance to investigate shrews to understand their role as potential zoonotic reservoirs in the transmission and harbouring of viruses that may be pathogenic to humans.

## Supplementary Material

250606_Supplementary_Material_veaf038

Virus_Evolution_Insectivore_suppl_veaf038

## Data Availability

All the sequence data generated in this study is available in NCBI GenBank with accession numbers ON720814–ON720835 and PP578938–PP578951.
