# Supplementary material for: Hantavirus co-circulation in common shrews (Sorex araneus) in Sweden
Source: Virus Evol. 2025 May 28;11(1):veaf038. doi: 10.1093/ve/veaf038 (PMC12202747; doi:10.1093/ve/veaf038)
Supplement: 250606_Supplementary_Material_veaf038 [file 250606_supplementary_material_veaf038.pdf]

Supplementary Material

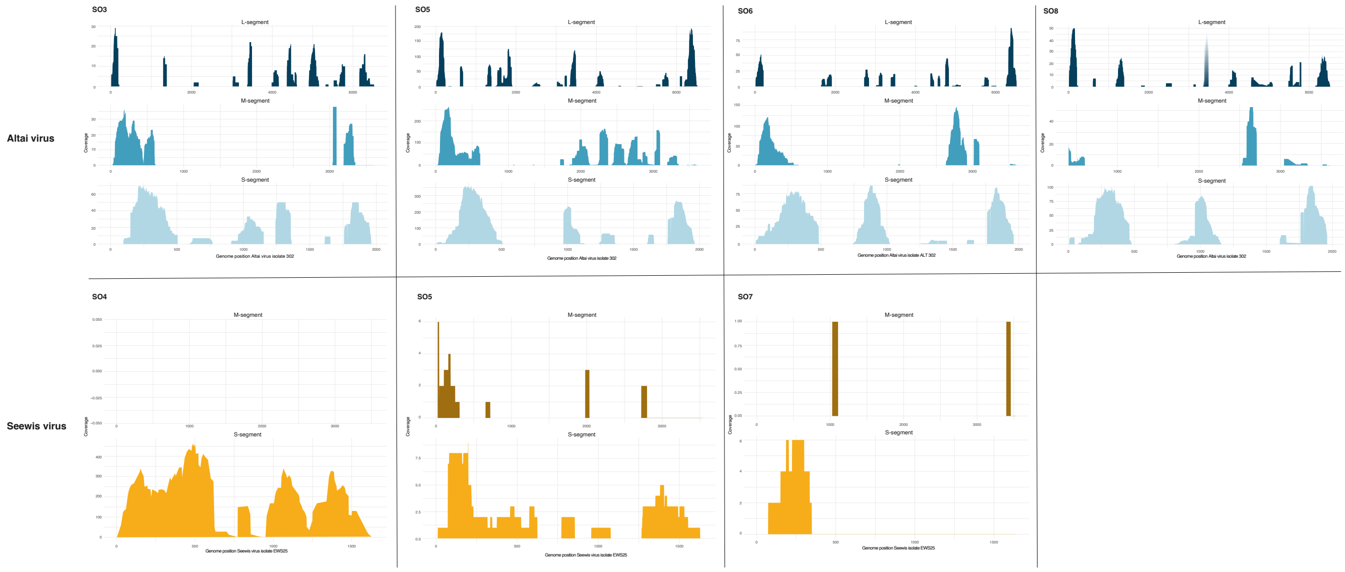

Supplementary Figure S1. Plots depicting the read coverage of Altai and Seewis reference sequences for each library. Russian Altai isolate 302 was used as a reference and is highlighted in blue. Reads mapped to the Finnish Seewis EWS25 isolate are highlighted in yellow. No full-length reference sequence is available for the Seewis virus L-segment.

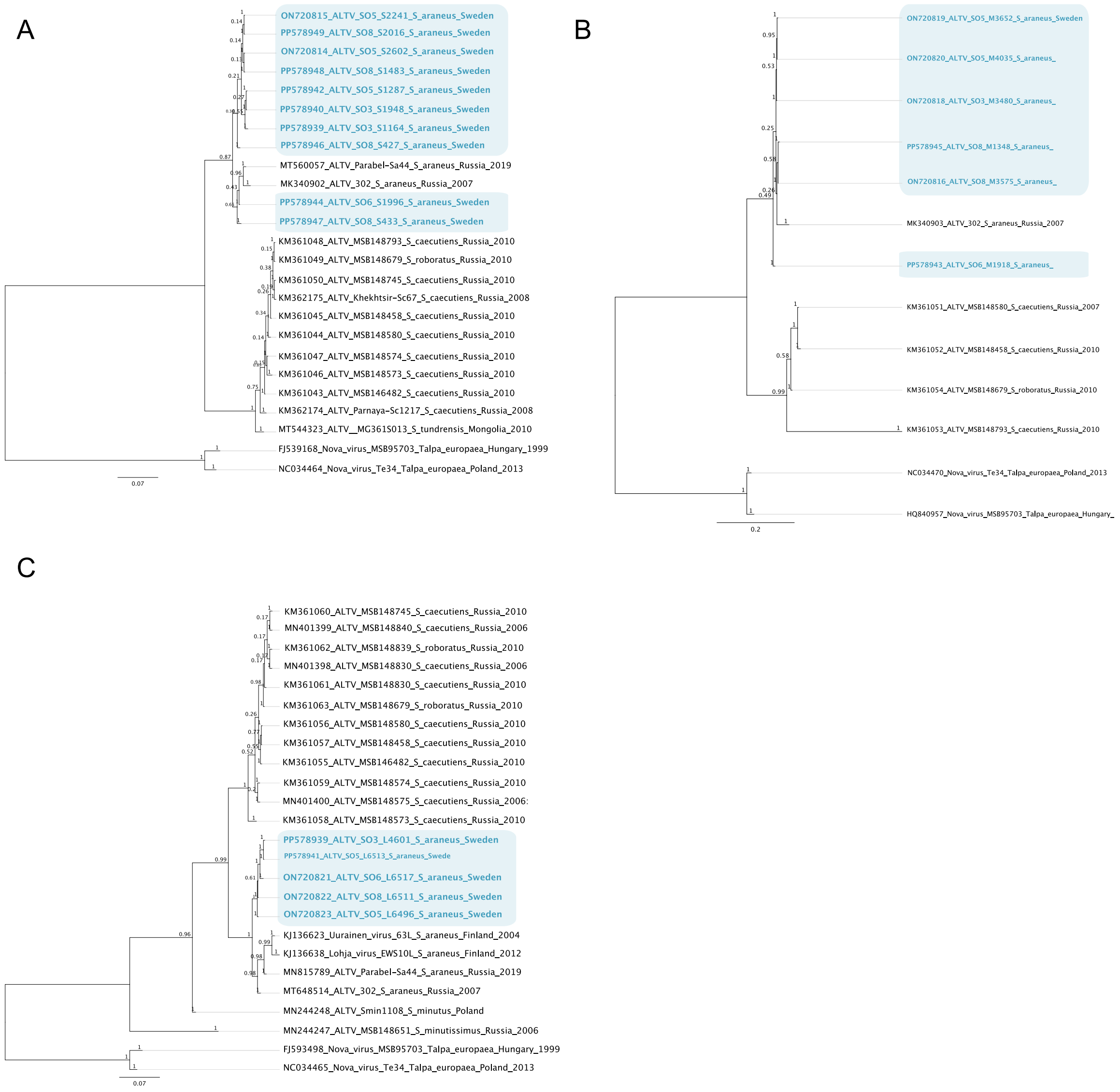

Supplementary Figure S2. Bayesian phylogenetic trees of Swedish Altai virus (ALTV) based on amino acid alignments of the (A) S-segment, (B) M-segment, and (C) L-segment. Sequences generated in this study are highlighted in blue. The trees include both full-length and partial sequences to represent the available diversity Phylogenetic reconstruction was performed using MrBayes under the WAG+I+G4 model. Posterior probability values are indicated at nodes, and scale bars represent amino acid substitutions per site.

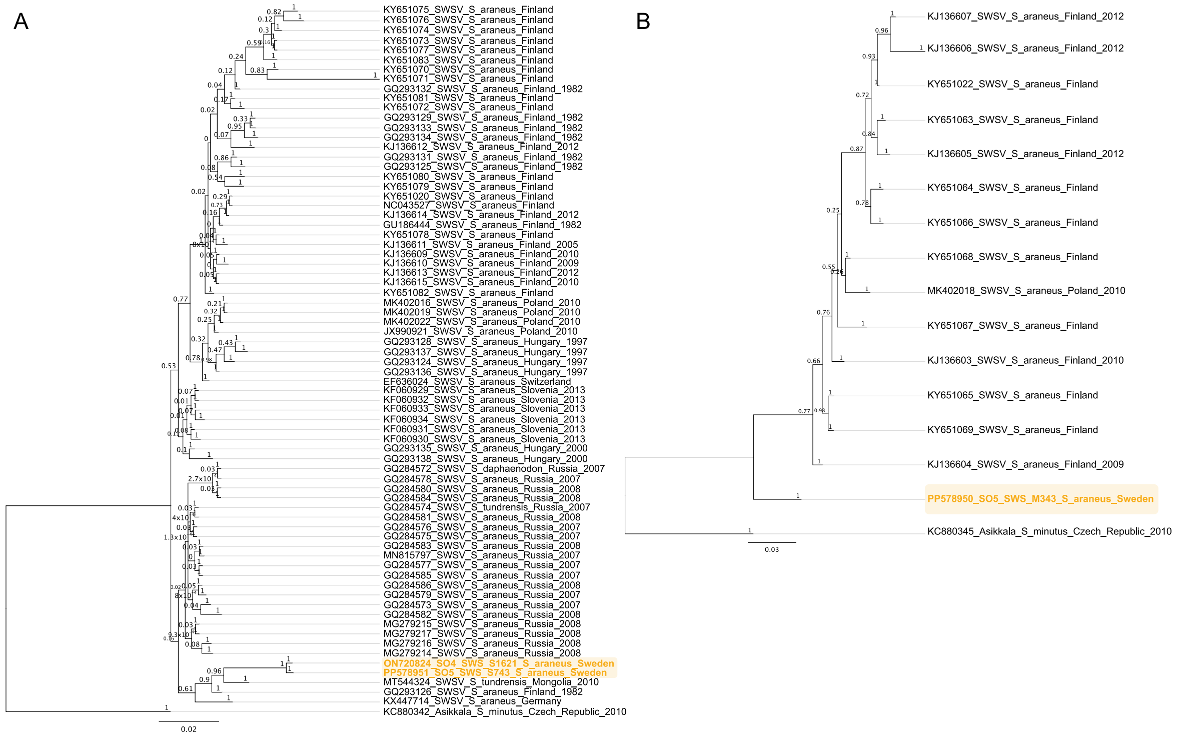

Supplementary Figure S3. Bayesian phylogenetic trees of Swedish Seewis virus (SWSV) based on amino acid alignments of the (A) S-segment and (B) M-segment. Swedish sequences are highlighted in yellow. The trees include both full-length and partial sequences to represent the available diversity. Phylogenetic analyses were performed using MrBayes under the WAG+I+G4 model. Posterior probability values

are indicated at nodes, and scale bars represent amino acid substitutions per site.

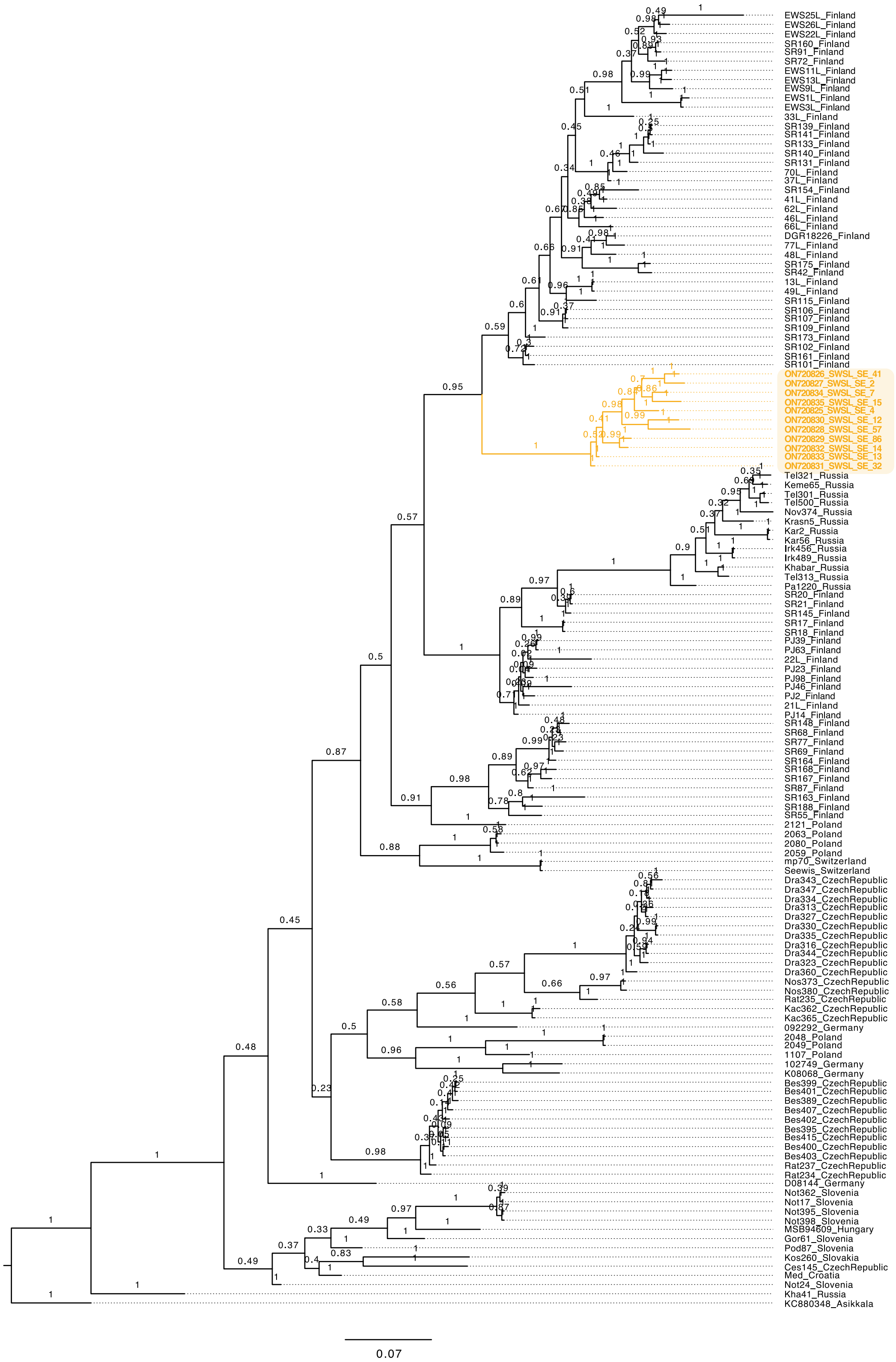

Supplementary Figure S4. Phylogenetic tree of partial L-segment of Swedish SWSV together with other partial L-sequences available at Genbank.

Supplementary Table S1. Sample information from animal samples used in this study.

**Area**	**Collection date**	***Collected from species***	**Sample no.**	**Pool**	**Congtigs for hantavirus**	**Hantavirus PCR**
Grimsö	2016-09-14	*Sorex araneus*	1911	SO1	None	
Grimsö	2016-09-14	*Sorex araneus*	1917	SO1		
Grimsö	2016-09-15	*Sorex araneus*	1935	SO1		Pos/Sequence
Grimsö	2016-09-15	*Sorex araneus*	1963	SO1		
Grimsö	2016-09-15	*Sorex araneus*	1973	SO1		
Grimsö	2016-09-15	*Sorex araneus*	1981	SO1		Pos/sequence
Grimsö	2016-09-15	*Sorex araneus*	1998	SO1		
Grimsö	2016-09-15	*Sorex araneus*	2021	SO1		Pos/Sequence
Grimsö	2016-09-15	*Sorex araneus*	2049	SO1		
Grimsö	2016-09-14	*Sorex araneus*	1930	SO1		
Grimsö	2017-10-3	*Sorex Araneus*	1864	SO1		
Grimsö	2016-09-15	*Sorex araneus*	2073	SO2	None	Pos/Sequence
Grimsö	2016-09-15	*Sorex araneus*	2088	SO2		
Grimsö	2016-09-20	*Sorex araneus*	2222	SO2		Pos/Sequence
Grimsö	2016-09-20	*Sorex araneus*	2260	SO2		
Grimsö	2016-09-20	*Sorex araneus*	2311	SO2		
Grimsö	2016-09-20	*Sorex araneus*	2360	SO2		
Grimsö	2016-09-20	*Sorex araneus*	2421	SO2		Pos/Sequence
Grimsö	2016-09-15	*Sorex araneus*	1940	SO2		
Grimsö	2016-09-15	*Sorex araneus*	1968	SO2		
Grimsö	2016-09-15	*Sorex araneus*	2042	SO2		
Grimsö	2016-09-15	*Sorex araneus*	2109	SO2		
Grimsö	2016-09-21	*Sorex araneus*	2626	SO3	Altai	

Grimsö	2016-09-21	*Sorex araneus*	2633	SO3		
Grimsö	2016-09-22	*Sorex araneus*	2806	SO3		
Grimsö	2016-09-22	*Sorex araneus*	2860	SO3		Pos/Sequence
Grimsö	2016-09-22	*Sorex araneus*	2912	SO3		
Grimsö	2015-04-23	*Sorex araneus*	303	SO3		
Grimsö	2015-09-23	*Sorex araneus*	1302	SO3		
Grimsö	2017-05-11	*Sorex araneus*	1133	SO3		
Grimsö	2017-05-10	*Sorex araneus*	1020	SO3		
Grimsö	2016-09-15	*Sorex araneus*	2114	SO3		
Grimsö	2016-09-20	*Sorex araneus*	2316	SO3		
Sala	2016-09-13	*Sorex araneus*	505	SO4	Seewis	
Sala	2016-09-13	*Sorex araneus*	669	SO4		
Sala	2016-09-14	*Sorex araneus*	863	SO4		
Sala	2016-09-14	*Sorex araneus*	1076	SO4		
Sala	2016-09-15	*Sorex araneus*	1170	SO4		Pos/Sequence
Sala	2016-09-15	*Sorex araneus*	1176	SO4		Pos/Sequence
Sala	2016-09-15	*Sorex araneus*	1209	SO4		
Sala	2016-09-20	*Sorex araneus*	1516	SO4		
Sala	2017-04-19	*Sorex araneus*	40	SO4		
Sala	2017-04-28	*Sorex araneus*	462	SO4		
Sala	2017-09-19	*Sorex araneus*	663	SO4		
Sala	2016-09-15	*Sorex araneus*	1265	SO5	Seewis and Altai	
Sala	2016-09-20	*Sorex araneus*	1423	SO5		
Sala	2016-09-20	*Sorex araneus*	1436	SO5		
Sala	2016-09-20	*Sorex araneus*	1484	SO5		
Sala	2017-09-28	*Sorex araneus*	1485	SO5		
Sala	2016-09-21	*Sorex araneus*	1761	SO5		
Sala	2016-09-21	*Sorex araneus*	1769	SO5		
Sala	2016-09-20	*Sorex araneus*	1661	SO5		
Sala	2016-09-21	*Sorex araneus*	1891	SO5		
Sala	2016-09-22	*Sorex araneus*	2067	SO5		

Sala	2016-09-22	*Sorex araneus*	1914	SO5		
Grimsö	2017-09-21	*Sorex araneus*	2600	SO6	Altai	Pos/Sequence
Grimsö	2017-09-21	*Sorex araneus*	2607	SO6		
Grimsö	2017-09-27	*Sorex araneus*	2967	SO6		Pos
Grimsö	2017-09-27	*Sorex araneus*	3034	SO6		
Grimsö	2017-09-27	*Sorex araneus*	3124	SO6		
Grimsö	2017-09-28	*Sorex araneus*	3245	SO6		Pos/Sequence
Grimsö	2017-09-29	*Sorex araneus*	3354	SO6		
Grimsö	2017-09-29	*Sorex araneus*	3426	SO6		
Grimsö	2017-09-29	*Sorex araneus*	3432	SO6		
Grimsö	2016-09-13	*Sorex araneus*	1358	SO6		
Grimsö	2016-09-13	*Sorex araneus*	1380	SO6		
Grimsö	2016-09-13	*Sorex araneus*	1366	SO7	Seewis	
Grimsö	2016-09-13	*Sorex araneus*	1373	SO7		
Grimsö	2016-09-13	*Sorex araneus*	1468	SO7		
Grimsö	2016-09-13	*Sorex araneus*	1490	SO7		
Grimsö	2016-09-13	*Sorex araneus*	1573	SO7		
Grimsö	2016-09-13	*Sorex araneus*	1596	SO7		Pos
Grimsö	2016-09-13	*Sorex araneus*	1637	SO7		
Grimsö	2016-09-13	*Sorex araneus*	1644	SO7		
Grimsö	2016-09-13	*Sorex araneus*	1671	SO7		
Grimsö	2016-09-14	*Sorex araneus*	1713	SO7		
Grimsö	2016-09-14	*Sorex araneus*	1762	SO7		
Grimsö	2016-09-14	*Sorex araneus*	1925	SO8	Altai	
Grimsö	2017-05-10	*Sorex araneus*	1020	SO8		
Grimsö	2017-05-11	*Sorex araneus*	1077	SO8		
Grimsö	2017-09-19	*Sorex araneus*	2167	SO8		
Grimsö	2017-09-19	*Sorex araneus*	2198	SO8		
Grimsö	2017-09-19	*Sorex araneus*	2204	SO8		
Grimsö	2017-09-19	*Sorex araneus*	2237	SO8		
Grimsö	2017-09-20	*Sorex araneus*	2433	SO8		
Grimsö	2017-09-20	*Sorex araneus*	2439	SO8		

Grimsö	2017-09-20	*Sorex araneus*	2499	SO8		
Grimsö	2017-09-20	*Sorex araneus*	2505	SO8		
Grimsö	2015-09-18	*Sorex araneus*	1224	-		
Grimsö	2015-09-23	*Sorex araneus*	1365	-		
Sala	2017-09-20	*Sorex araneus*	721	-		
Sala	2017-09-21	*Sorex araneus*	858	-		
Sala	2017-09-21	*Sorex araneus*	814	-		
Grimsö	2017-09-19	*Sorex araneus*	2344	-		
Grimsö	2017-09-19	*Sorex araneus*	2356	-		
Grimsö	2017-09-19	*Sorex araneus*	2362	-		
Grimsö	2017-09-20	*Sorex araneus*	2427	-		
Grimsö	2017-09-20	*Sorex araneus*	2520	-		
Grimsö	2017-09-21	*Sorex araneus*	2553	-		
Grimsö	2017-09-21	*Sorex araneus*	2566	-		
Grimsö	2017-09-21	*Sorex araneus*	2588	-		
Sala	2017-09-27	*Sorex araneus*	1159	-		
Sala	2017-09-27	*Sorex araneus*	1280	-		
Sala	2017-09-27	*Sorex araneus*	1304	-		
Sala	2017-09-28	*Sorex araneus*	1330	-		
Sala	2017-09-28	*Sorex araneus*	1459	-		
Bogesund	2015-10-01	*Sorex araneus*	1483	-		
Bogesund	2015-10-01	*Sorex araneus*	1528	-		
Bogesund	2015-10-03	*Sorex araneus*	1679	-		
Bogesund	2016-09-30	*Sorex araneus*	1733	-		
Bogesund	2016-09-30	*Sorex araneus*	1766	-		

Supplementary table S2. Overview of the partial viral sequences included in the phylogenetic analyses. The table lists each sequence with its corresponding virus, accession number, sequence name, origin country, final alignment length, and sequence length after trimming. The final alignment length refers to the length of the alignment after trimming and gap removal as used in the phylogenetic reconstructions.

Virus	Accession no.	Name	Origin country	Segment	Final alignment length (nt)	Sequence length (nt)
ALTV	PP578942	SO5_S1287	Sweden	S	930	686
ALTV	PP578939	SO3_S1164	Sweden	S	930	705
ALTV	PP578946	SO8_S427	Sweden	S	930	338
ALTV	PP578947	SO8_S433	Sweden	S	930	433
ALTV	KM361046	MSB148573	Russia	S	930	704
ALTV	KM361057	MSB148458	Russia	S	930	841
ALTV	KM361050	MSB148745	Russia	S	930	698
ALTV	KM362175	Kehkhstir-Sc67	Russia	S	930	553
ALTV	KM362174	Parnaya-Sc1217	Russia	S	930	831
ALTV	PP578943	SO6_M1918	Sweden	M	2161	1619
ALTV	PP578945	SO8_M1348	Sweden	M	2161	1348
ALTV	KM361051	MSB148580	Russia	M	2161	568
ALTV	KM361052	MSB148458	Russia	M	2161	556
ALTV	KM361053	MSB148793	Russia	M	2161	464
ALTV	KM361054	MSB148679	Russia	M	2161	460
ALTV	KJ136623	Uurainen_virus_63L	Finland	L	2375	282
ALTV	KJ136638	Lohja_virus_EWS10L	Finland	L	2375	282
ALTV	MN815789	Parabel-Sa44	Russia	L	2375	282
ALTV	KM361058	MSB148573	Russia	L	2375	408
ALTV	KM361059	MSB148574	Russia	L	2375	408
ALTV	MN401400	MSB148575	Russia	L	2375	408
ALTV	KM361060	MSB148745	Russia	L	2375	408
ALTV	MN401399	MSB148840	Russia	L	2375	408
ALTV	KM361062	MSB148839	Russia	L	2375	423
ALTV	MN401398	MSB148830	Russia	L	2375	420
ALTV	MN244247	MSB148651	Russia	L	2375	358
ALTV	MN244248	Smin1108	Poland	L	2375	769
SWSV	MG279214	Parnaya-Sa1197	Russia	S	786	510
SWSV	MK402016	Sa10-1	Poland	S	786	619
SWSV	MK402019	Sa10-5	Poland	S	786	650
SWSV	JX990921	1107	Poland	S	786	689
SWSV	GQ293124	MSB95462	Hungary	S	786	643
SWSV	GQ293128	MSB95468	Hungary	S	786	438
SWSV	GQ293137	MSB95464	Hungary	S	786	498
SWSV	GQ293125	DGR18228	Finland	S	786	394
SWSV	GQ293131	DGR18279	Finland	S	786	394
SWSV	GQ293129	DGR18891	Finland	S	786	394
SWSV	GQ293134	DGR18889	Finland	S	786	394
SWSV	GQ293132	DGR18874	Finland	S	786	394
SWSV	GQ293133	DGR18887	Finland	S	786	394
SWSV	GQ293135	MSB94615	Hungary	S	786	394
SWSV	GQ293138	MSB94609	Hungary	S	786	642
SWSV	GQ293126	DGR18207	Finland	S	786	394
SWSV	PP578951	SO5 S743	Sweden	S	786	670
SWSV	MK402018	Sa10-5	Poland	M	1187	794
SWSV	KJ136605	EWS13	Finland	M	1187	847
SWSV	KJ136606	EWS26	Finland	M	1187	970
SWSV	PP578950	SO3 M343	Sweden	M	1187	343
