# Supplementary material for: Hantavirus co-circulation in common shrews (Sorex araneus) in Sweden
Source: Virus Evol. 2025 May 28;11(1):veaf038. doi: 10.1093/ve/veaf038 (PMC12202747; doi:10.1093/ve/veaf038)
Supplement: Virus_Evolution_Insectivore_suppl_veaf038 [file virus_evolution_insectivore_suppl_veaf038.docx]

**Supplementary Material**

**Supplementary Figure S1.** Plots depicting the read coverage of Altai and Seewis reference sequences for each library. Russian Altai isolate 302 was used as a reference and is highlighted in blue. Reads mapped to the Finnish Seewis EWS25 isolate are highlighted in yellow. No full-length reference sequence is available for the Seewis virus L-segment.

**Supplementary Figure S2**. Bayesian phylogenetic trees of Swedish Altai virus (ALTV) based on amino acid alignments of the (A) S-segment, (B) M-segment, and (C) L-segment. Sequences generated in this study are highlighted in blue. The trees include both full-length and partial sequences to represent the available diversity Phylogenetic reconstruction was performed using MrBayes under the WAG+I+G4 model. Posterior probability values are indicated at nodes, and scale bars represent amino acid substitutions per site.

**Supplementary Figure S3**. Bayesian phylogenetic trees of Swedish Seewis virus (SWSV) based on amino acid alignments of the (A) S-segment and (B) M-segment. Swedish sequences are highlighted in yellow. The trees include both full-length and partial sequences to represent the available diversity. Phylogenetic analyses were performed using MrBayes under the WAG+I+G4 model. Posterior probability values are indicated at nodes, and scale bars represent amino acid substitutions per site.

**Supplementary Figure S4.** Phylogenetic tree of partial L-segment of Swedish SWSV together with other partial L-sequences available at Genbank.

**Supplementary Table S1.** Sample information from animal samples used in this study.

**Supplementary table S2.** Overview of the partial viral sequences included in the phylogenetic analyses. The table lists each sequence with its corresponding virus, accession number, sequence name, origin country, final alignment length, and sequence length after trimming. The final alignment length refers to the length of the alignment after trimming and gap removal as used in the phylogenetic reconstructions.
